# Highly Efficient Adsorption of Phenylethanoid Glycosides on Mesoporous Carbon

**DOI:** 10.3389/fchem.2019.00781

**Published:** 2019-11-14

**Authors:** Helin Xu, Wenjing Pei, Xueqin Li, Jinli Zhang

**Affiliations:** ^1^School of Chemistry and Chemical Engineering/Key Laboratory for Green Processing of Chemical Engineering of Xinjiang Bingtuan, Shihezi University, Shihezi, China; ^2^Key Laboratory for Green Chemical Technology of Ministry of Education, School of Chemical Engineering and Technology, Tianjin University, Tianjin, China

**Keywords:** *Cistanche tubulosa*, acteosides, echinacosides, phenylethanoid glycosides, mesoporous carbon, kinetic model, isothermal model

## Abstract

PhGs are the major active compounds of *Cistanche tubulosa*, and it is extremely desirable for obtaining high purification of PhGs by adsorption from their extracts. To explore highly efficient adsorption of PhGs, a novel adsorption material for the efficient separation and purification of phenylethanoid glycosides (PhGs) from *Cistanche tubulosa* was explored. The three mesoporous carbons of ordered mesoporous carbon (CMK-3), disordered mesoporous carbon (DMC), and three-dimensional cubic mesoporous carbon (CMK-8) were compared for adsorption of PhGs. Meanwhile, adsorption isotherms, adsorption kinetics, and the optimization of adsorption conditions were investigated. The results indicated that CMK-3 showed the highest adsorption capacity of 358.09 ± 4.13 mg/g due to its high specific surface area, large pore volume and oxygen-containing functional groups. The experimental data can be accurately described using the Langmuir model and pseudo-second-order model. The intra-particle diffusion model suggested that the rate-limiting steps of adsorption were intra-particle diffusion.

## Introduction

*Cistanche tubulosa* was an Orobanchaceae parasitic plant (Li et al., 2016; Wang X. et al., 2017), and it mainly grown on the roots of Tamarix plants and Calotropis species (Zhang W. et al., 2016; Yan et al., 2017). *Cistanche tubulosa* was originally recorded in Shen Nong's Chinese Materia Medica in ca. 100 B.C. The growth and cultivation of *Cistanche tubulosa* required severe environmental conditions, and it was widely planted in arid lands and deserts of the northern hemisphere, such as the provinces of Xinjiang, Inner Mongolia, Gansu, Qinghai, and the Ningxia Autonomous Region in China (You et al., 2016). *Cistanche tubulosa* was a precious Chinese tonic herb which had the functions of nourish the kidney, anti-aging, boosts the essence of blood, and moistens the large intestines to free stool (Gu et al., 2016; Shimada et al., 2017; Cui et al., 2018), and it has been reputation as “Ginseng of the Deserts” (Song et al., 2016; Wang et al., 2018). *Cistanche tubulosa* was officially recorded in the Chinese Pharmacopeia as the authentic source of *Cistanches Herba* (Chinese name: Roucon-grong) from 2005 edition (Wang T. et al., 2016; Pei et al., 2019).

Previous study had revealed several main chemical constituents of *Cistanche tubulosa*, including PhGs, iridoids, and polysaccharides (Li et al., 2018a). The structures of PhGs were mainly composed of cinnamic acid and phenylethyl alcohol that were attached to a β-glucopyranose through ester and glycosidic linkages (Luo et al., 2010), and PhGs have been regarded as the major active components of *Cistanche tubulosa* possessing various pharmacological activities (Liao et al., 2018). The study shown that PhGs had a variety of medicinal properties, such as neuroprotection, immune regulation, anti-inflammatory, liver protection and antioxidant (Aiello et al., 2015; Shiao et al., 2017; Wu et al., 2018, 2019). According to phytochemical evaluations, PhGs such as echinacoside, acteoside were considered to be main active components and markers of *Cistanche tubulosa* (Li et al., 2017b), which were usually chosen as marker compounds for the quality evaluation of *Cistanche tubulosa* and the species of *Cistanche* were distinguished through these compounds. PhGs were naturally occurring water-soluble compounds because it had many hydroxyl groups and phenolic hydroxyl groups in the molecule. Thus, the PhGs can be separated from *Cistanche tubulosa* in an aqueous solution.

Many methods for the separation and purification of natural products have been developed including adsorption (Liu et al., 2016), membrane separation (Zhang et al., 2018b; Li et al., 2019) and solvent extraction, and so on (Li et al., 2015a,b; Wang S. et al., 2016; Zhang H. et al., 2016). However, membrane separation and solvent extraction were not suitable for large-scale preparation and they were difficult to achieve high recovery of the products (Zhang et al., 2018a). Adsorption was one of the most widely adopted methods for the separation natural products (Wang S. et al., 2016; Konggidinata et al., 2017). Owing to its unique and tunable pore structures, high surface areas and mechanical stability, mesoporous carbons (pore size between 2 and 50 nm) have been proven to be a kind of efficient adsorbents for adsorptive natural products. The study shown that mesoporous carbons were more suitable for adsorbing macromolecules, such as mesoporous carbons have been used by Qin et al. to enrichment of chlorogenic acid from eucommia ulmoides leaves (Qin et al., 2018). Li et al. synthesized two mesoporous carbons via a hydrothermal treatment approach, and evaluated adsorption performance of two mesoporous carbons for berberine hydrochloride and matrine from water (Li et al., 2018b). It was considered to be kind of promising material as a highly efficient adsorbent (Zhang et al., 2013; Tian et al., 2015; Zhou et al., 2016). Additionally, mesoporous carbons also have been applied on adsorptive removal of aromatic compounds, dyes, and heavy metals from wastewater (Kong et al., 2016). In previous published works, Liu et al. used a macroporous resin to adsorb PhGs from *Cistanche tubulosa*, and the purity of the PhGs increased but the adsorption capacity and desorption rate were low. Compared with macroporous resin, mesoporous carbons had the characteristics of large specific surface area, suitable pore size, and a high pore volume. Therefore, mesoporous carbon was considered to be a highly efficient adsorbent for PhGs. In this study, the three kinds of mesoporous carbon were selected as adsorbent for separation and purification of PhGs from *Cistanche tubulosa*.

This work main objective was to explore the adsorption performance of CMK-3 for separation and purification PhGs from *Cistanche tubulosa*. The effects of different concentrations, pH and temperature on the adsorption performance of CMK-3 were investigated and optimal adsorption conditions of PhGs were screened out. Mesoporous carbons were characterized by FT-IR, BET, TEM, and TGA, adsorption isotherms and kinetics were performed and analyzed in detail.

## Experiments

### Materials and Reagents

*Cistanche tubulosa* stem was purchased from Congrongtang Biological Technology Co., Ltd. (Xinjiang). The standards of enchinacoside (purities ≥ 98%) and acteoside (purities ≥ 98%) were purchased from Sunny Biotech Co., Ltd. (Shanghai). Acetonitrile, methanol and acetic acid of HLPC were purchased from Thermo Fisher Scientific Co., Ltd. (Shanghai). The ethanol of analytical grade was purchased from Yongsheng Fine Chemical Co., Ltd. (Tianjin). Ordered mesoporous carbon (CMK-3), disordered mesoporous carbon (DMC) and three-dimensional cubic ordered mesoporous carbon (CMK-8) were purchased from Xianfeng Nano Material Technology Co., Ltd. (Nanjing).

### Characterization

The morphology and microstructures of the prepared samples were investigated using Transmission electron microscopy (TEM, Tecnai G2 F20) operated at 200 KV. The TEM samples were prepared under ambient conditions by depositing droplets of the ethanol solution with the mesoporous materials onto carbon films supported by Cu grids. Generally, a light source with a shorter wavelength was selected to increase the resolution of the microscope, and the structure of the mesoporous carbons can be clearly observed. The surface functional groups were qualitatively measured by Fourier transform infrared spectoscopy (FT-IR, AVATAR360) using the interaction between infrared radiation and matter molecules. FT-IR use attenuated total reflection method test, the conditions was step size of 2 cm^−1^ and scanning range was 4,000–400 cm^−1^. The physical structure data such as the specific surface area, pore size and pore volume of the mesoporous carbons calculated by Brunauer-Emmett-Teller (BET, ASAP 2460). The procedure for the adsorbent was as follows: mesoporous carbons were degassed at 60°C for 12 h, and the N_2_ adsorption-desorption curves were tested at −196°C to calculate the specific surface area, pore size and pore volume of the mesoporous carbon. Thermo gravimetric analyzer (TGA, STA 449 F3) is an instrument that it uses the thermo gravimetric to detect the temperature-mass relationship of a substance, and TGA measures the mass of a substance as a function of temperature under program temperature control. TGA data was obtained using a TGA in the temperature ranging from 30 to 800°C at a heating rate of 10°C/min under air atmosphere.

### HPLC Analysis

The content of echinacoside and acteoside was detection by high performance liquid chromatography (HPLC, Waters Co., USA). The system included an autosampler, high pressure pump and ultraviolet (UV) detector. The analysis was conducted on a symmetry C18 column (100Å, 5 μm, 4.6 × 250 mm). HPLC used gradient elution method to separate and detect samples. The volume of injection loop was 10 μm, the column temperature was 30°C, detection wavelength of UV spectrophotometer was 330 nm, the flow rate was 1 ml/min and the mobile phase was (A) acetonitrile and (B) acetic acid/water (1:44, v/v).

### Adsorption Equilibrium

The optimization experiment of adsorption condition for CMK-3 has been carried out using a mixture of acteoside and enchanoside and under the optimal conditions, the crude extract of *Cistanche tubulosa* was carried out on adsorption cycle experiment and all adsorption experiments were repeatedly carried out at least 3. In the same batch of experiments, mesoporous carbons of CMK-8 and DMC were run in parallel with the CMK-3. The three kinds of mesoporous carbon (CMK-3, DMC and CMK-8) each 10 mg were added to the three bottles, respectively. Then 15 mL sample solution with initial concentration of C_0_ (mg/mL) was added to bottle. The bottle was placed in constant temperature shaker of 30°C for 24 h until the adsorption equilibrium was reached. Then 1 ml of adsorption solution was filtered through a 0.22 μm filter and the equilibrium concentration C_e_ (mg/mL) of the sample solution was determined by HPLC.

### Desorption Experiment

Then the desorption experiment of mesoporous carbon were carried out. The adsorbed mesoporous carbon under 15 mL of methanol/acetic acid (9:1, v/v) mixed solution, which was placed in the water bath of ultrasonic for 1 h at 30°C. The obtained desorption solution was filtered by a 0.22 filter before analyzing by HPLC.

The adsorption capacity q_e_ (mg/ml) was evaluated as follows:

(1) $${\text{q}}_{\text{e}}=({\text{C}}_{0}-{\text{C}}_{\text{e}})\cdot \text{v}/\text{w}$$

where V is the volume of the solution (mL) and W is the weight of the mesoporous carbons (g).

## Results and Discussion

### Characterization

**Figure 1:**
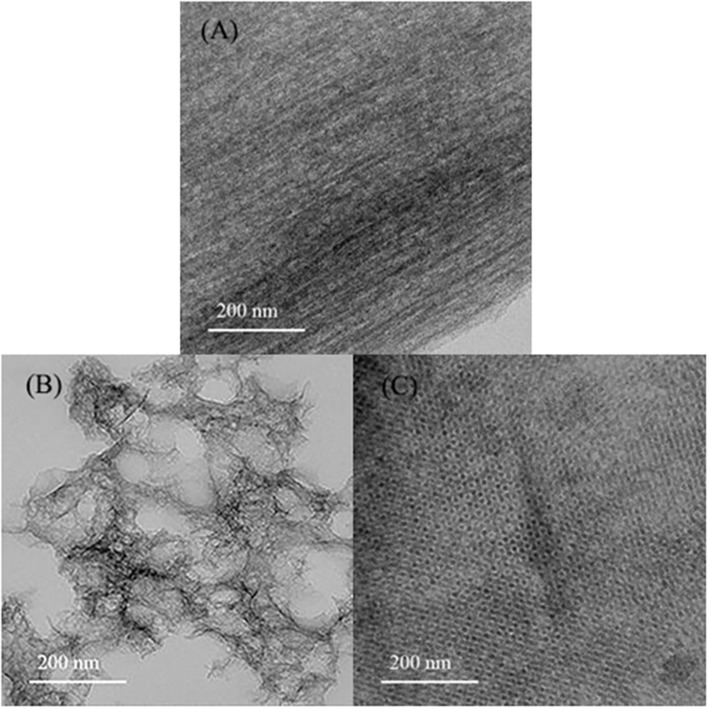
TEM of **(A)** CMK-3, **(B)** DMC, and **(C)** CMK-8.

Figure 1 showed a TEM of the three kinds of mesoporous carbons. DMC was a disordered porous network, CMK-8 was a network structure of three-dimensional porous, and CMK-3 was a clearly striped structure with ordered one-dimensional pore, which was similar to the reported results (Wang et al., 2006; Luo et al., 2010).

**Figure 2:**
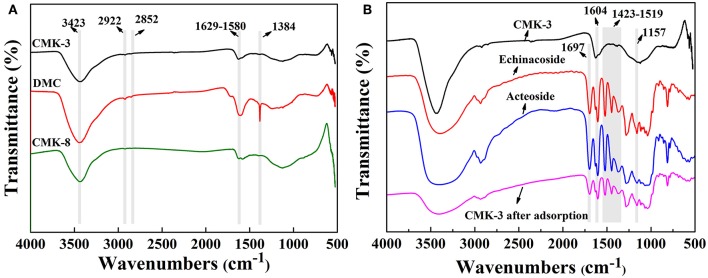
FT-IR spectra of **(A)** CMK-3, DMC, CMK-8, and **(B)** CMK-3 before and after adsorption, Echinacoside and Acteoside.

Figure 2 showed the FT-IR spectrum of the mesoporous carbons (CMK-3, DMC, and CMK-8) and FT-IR spectrum before and after CMK-3 adsorption. It can be seen from in Figure 2A that the functional groups on the surfaces of the mesoporous carbons were mainly oxygen-containing groups. The overall shapes of the spectra for the three kinds of mesoporous carbons were similar. The mesoporous carbons showed a peak band at 3,423 cm^−1^ referring to stretching vibration band of O-H. The bands in the region of 1,580 and 1,629 cm^−1^ correspond to stretching vibrations of the carbonyl and carboxyl C = O. Additionally, the peak occurring at 1,384 cm^−1^ was found to be stretching vibrations of alcoholic C-O and the tensile vibration at 2,922 and 2,852 cm^−1^ are correspond to the C-H on methylene and methyl groups, respectively. This indicated that the oxygen-containing groups existing on the surfaces of the mesoporous carbons might lead to a weak chemical interaction between PhGs molecules and the mesoporous carbons.

Figure 2B shows the FT-IR spectra of CMK-3 before and after adsorption, acteoside and enchanoside. The characteristic peak at 1,697 cm^−1^ derived from the C = C of olefin in acteoside and enchanoside, while the bands in the region of 1,519–1,423 cm^−1^ corresponded to stretching vibration peak of the aromatic ring C = C in acteoside and enchanoside. The tensile vibration at the 1,604 cm^−1^ was the C = O bond and the peak at 1,157 cm^−1^ caused by the stretching vibration of the ether bond in acteoside and enchanoside. Compared with the FT-IR spectrum of CMK-3 before adsorption, the FT-IR spectrum of CMK-3 after adsorption appeared the new peaks, which belonged to the characteristic peak of acteoside and enchanoside.

**Figure 3:**
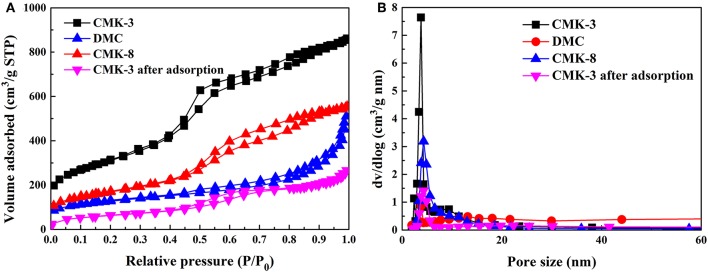
**(A)** N_2_ adsorption-desorption isotherms and **(B)** Pore size distributions of the mesoporous carbons, and CMK-3 after adsorption.

The N_2_ adsorption-desorption isotherms was an important parameter for adsorption of PhGs on CMK-3 and comparison of adsorbent structure. Figure 3 showed the N_2_ adsorption-desorption isotherms of CMK-3, CMK-8, DMC, and CMK-3 after PhGs adsorption, respectively. As can be seen from the Figure 3, the isotherm of the mesoporous carbons were similar to type-IV isotherm that this type of isotherm was predominantly mesoporous, in which the range of pore size was between 2 and 50 nm (Sanz Pérez et al., 2019). The gap between adsorption and desorption isotherm was referred to hysteresis loop that caused by capillary condensation reaction. For capillary condensation reactions, capillary condensation occurs first in the smallest pores (Barsotti et al., 2016). This shows that CMK-3 had a smaller mesopore than does DMC and CMK-8, which was consistent with the results of Table 1. The isotherm of CMK-3 exhibits an H_1_ hysteresis loop that was indicative of the rapid pore filling associated with capillary condensation and the pore structure of CMK-3 was reasonably orderly. The isotherm of DMC exhibits H_3_ hysteresis loop, this type of hysteresis had disordered pores due to network of pores that caused an undefined structure of porous adsorbent. CMK-8 isotherms exhibit an H_2_ hysteresis loop, indicating that pore structure was complicated and pore size distribution was uneven.

**Table 1:** BET characterization data of mesoporous carbons.

**Mesoporous carbon**	**Hysteresis curve**	**Pore size (nm)**	**Pore volume (cm^**3**^/g)**	**Specific surface area (m^**2**^/g)**
CMK-3	H_1_	4.31	1.32	1,098.02
DMC	H_3_	9.86	0.70	430.42
CMK-8	H_2_	5.18	0.85	596.00
CMK-3 after adsorption	H_1_	6.03	0.42	227.75

The N_2_ adsorption-desorption isotherms of CMK-3 was compared before and after adsorption of PhGs. The isotherm of the CMK-3 after adsorption was also similar to type-IV isotherm in Figure 3B. It indicated that the CMK-3 maintained their mesoporous structure after the adsorption. As can be seen from Table 1, the specific surface and pore volume of CMK-3 after adsorption exhibited a marked decrease, the specific surface area of CMK-3 before and after adsorption decreased from 1,098.02 to 227.75 m^2^/g, and the pore volume of that reduced from 1.32 to 0.42 cm^3^/g. It indicated that PhGs molecules were adsorbed on CMK-3.

Table 1 summarized the BET specific surface area, pore volume and pore size of the four samples. The BET surface areas of CMK-3, DMC and CMK-8 were 1,098.02, 430.42, and 596.00 m^2^/g and the pores volume were 1.32, 0.70, and 0.85 m^3^/g, respectively. The pore size of CMK-3 was 4.31 nm, lower than that of CMK-8 (9.58 nm) and DMC (5.18 nm). It can be seen that the pore volume and specific surface area follows the order: CMK-3 >CMK-8 >DMC, while pore size follows the order: DMC >CMK-8 >CMK-3.

**Figure 4:**
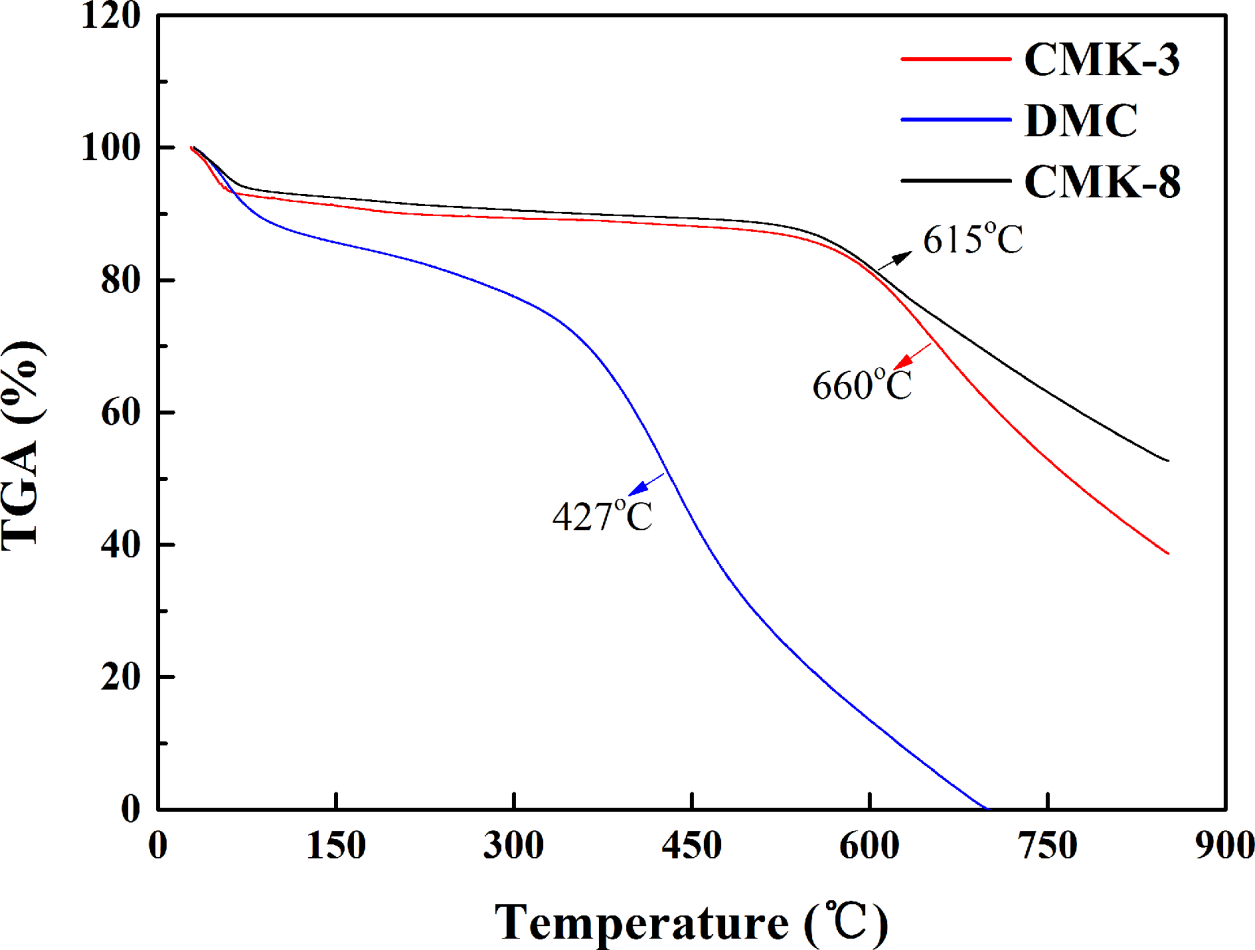
TGA curves of three kinds of mesoporous carbons.

Figure 4 shows the TGA curves of the three kinds of mesoporous carbons (CMK-3, CMK-8, and DMC). As can be seen from the Figure 4, the three kinds of mesoporous carbons all have two distinct stages of mass loss: the first stage of mass loss was due to the evaporation of moisture in the mesoporous carbons before 100°C, the second mass loss stage of CMK-3, DMC and CMK-8 approximately occurs at 660, 427, and 615°C, respectively, which corresponds to the oxidative thermal decomposition of mesoporous carbons materials. It can be seen that the thermal decomposition temperature of CMK-3 was higher than CMK-3 and CMK-8, the thermal stability of CMK-3 was better than that of CMK-8 and DMC.

### Comparison of the Three Mesoporous Carbons Adsorption Performance

Because the three adsorbents are mainly composed of carbon, the adsorption interaction between PhGs and the mesoporous carbons was considered to be the same. Therefore, it was considered that the difference in adsorption performance was derived from the difference in the physical structure of mesoporous carbons. Table 2 shows the adsorption performance of the three kinds of mesoporous carbons. It can be seen from Table 2 that the three kinds of mesoporous carbons all can adsorb PhGs, and CMK-3 had the better adsorption performance than CMK-8 and DMC. This indicates that pore size of mesoporous carbons was larger than PhGs molecules, and pore size was not the main factor to affect the adsorption capacity for three mesoporous carbons. The adsorption capacity and desorption rates of CMK-3 were up to 189.37 mg/g and 94.96%, respectively. The adsorption capacity of CMK-3 was higher than that of CMK-8. Because the pore volume and specific surface area of CMK-3 were larger than DMC and CMK-8, it indicated that the pore volume and specific surface area was main factor to affect the adsorption performance.

**Table 2:** Adsorption performance of three kinds of mesoporous carbons.

**Mesoporous carbon**	**Adsorption quality (mg/g)**	**Desorption quality (mg/g)**	**Desorption rates (%)**
CMK-3	189.37 ± 4.52	179.86 ± 3.36	94.98 ± 1.34
DMC	80.25 ± 2.60	74.05 ± 0.62	92.28 ± 0.77
CMK-8	135.75 ± 5.41	128.08 ± 3.18	94.35 ± 1.16

### Optimization of Adsorption Conditions

The optimization of adsorption experiment for CMK-3 has been carried out using a mix of both acteoside and enchanoside. Temperature, pH and concentration were the main influencing factors of CMK-3 adsorption performance. Thus, the effects of these three influencing factors on CMK-3 adsorption performance were investigated.

### Effect of Sample Concentration on Adsorption Performance of CMK-3

**Figure 5:**
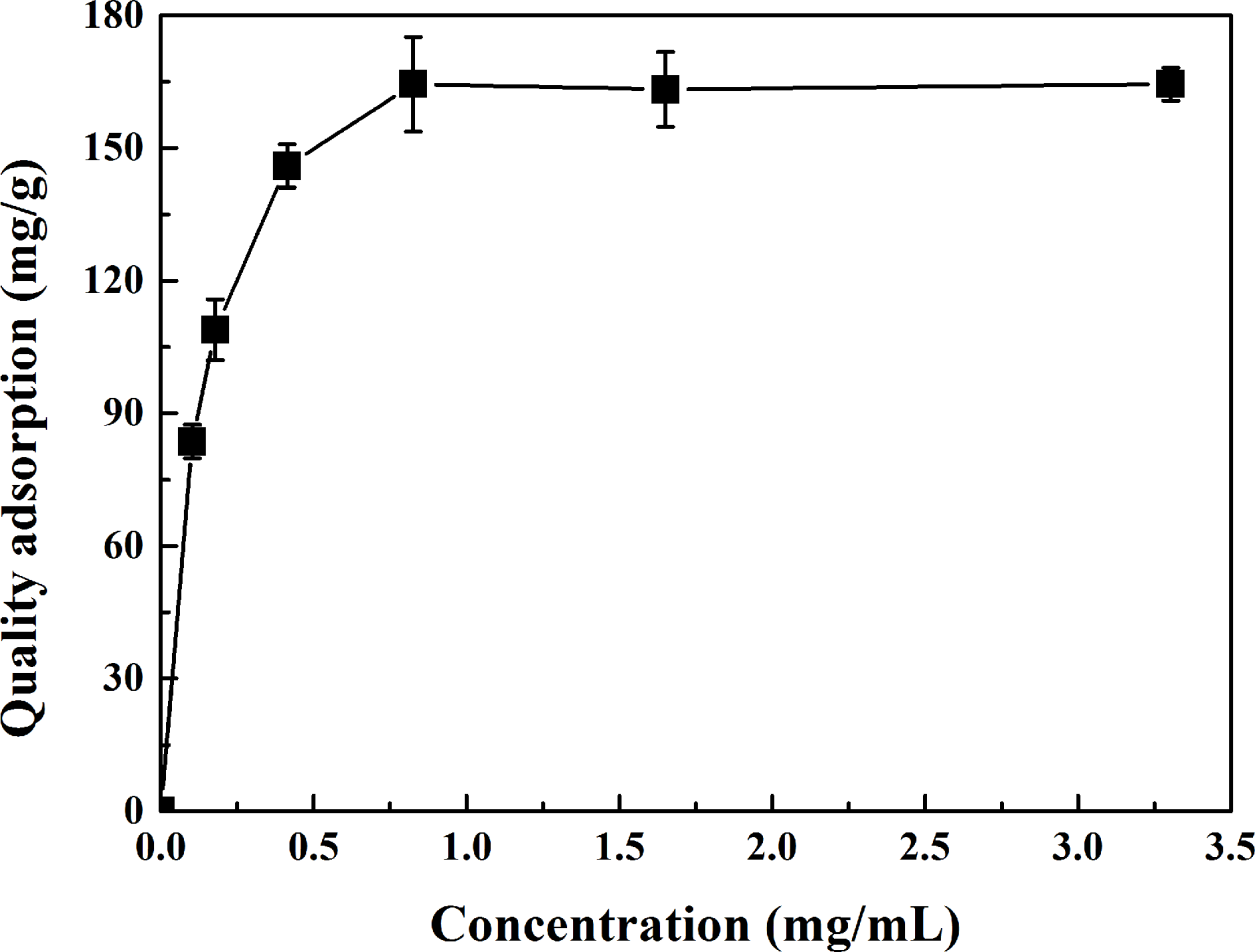
The effect of sample concentration on the adsorption of CMK-3.

The effect of sample concentration on the adsorption performance of CMK-3 was shown in Figure 5. With the concentration increases, the capacity of adsorption increases. The adsorption capacity no longer increases with the increase of the sample concentration when the sample concentration was reached at 0.41 mg/g. At low initial concentration of sample, the adsorbent active sites were sufficient for adsorption of relatively small amount of PhGs molecules. In contrast, at high initial concentration of sample, the fixed amount of active sites on the adsorbents was not able to adsorption increasing amount of PhGs molecules. Thus, the adsorption capacity of CMK-3 for PhGs molecules tends to be balanced.

### Effect of pH on Adsorption Performance of CMK-3

**Figure 6:**
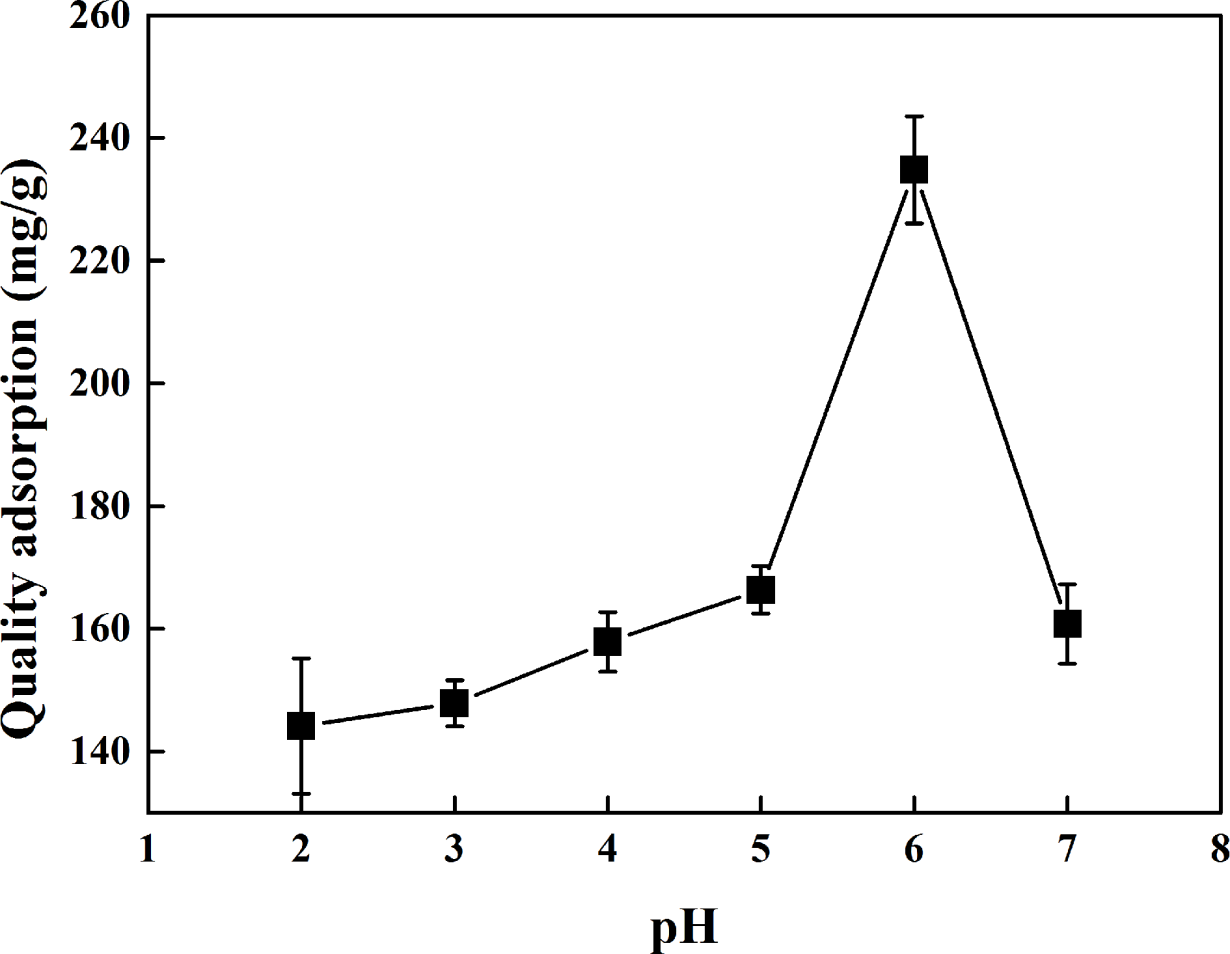
The effect of pH on the adsorption of CMK-3.

Figure 6 showed the effect of pH on the adsorption performance of CMK-3. It can be seen from Figure 6 that the optimal pH value was 6 for adsorption of PhGs by CMK-3. The reasons as follows: PhGs had a large number of phenolic hydroxyl groups and belonged to the weak acidic molecules, the different pH values affected the ionization and stability of the PhGs molecules. The ionization of phenolic hydroxyl groups would be inhibited at low pH value, while the stability of phenolic hydroxyl groups on PhGs would decrease at high pH value. The inhibited ionization of phenolic hydroxyl groups on PhGs resulted in a decrease in the electrostatic interactions between PhGs and CMK-3, decreasing the adsorption performance of CMK-3 for PhGs. Thus, the optimum pH was 6 for CMK-3 adsorption.

### Effect of Temperature on Adsorption Performance of CMK-3

**Figure 7:**
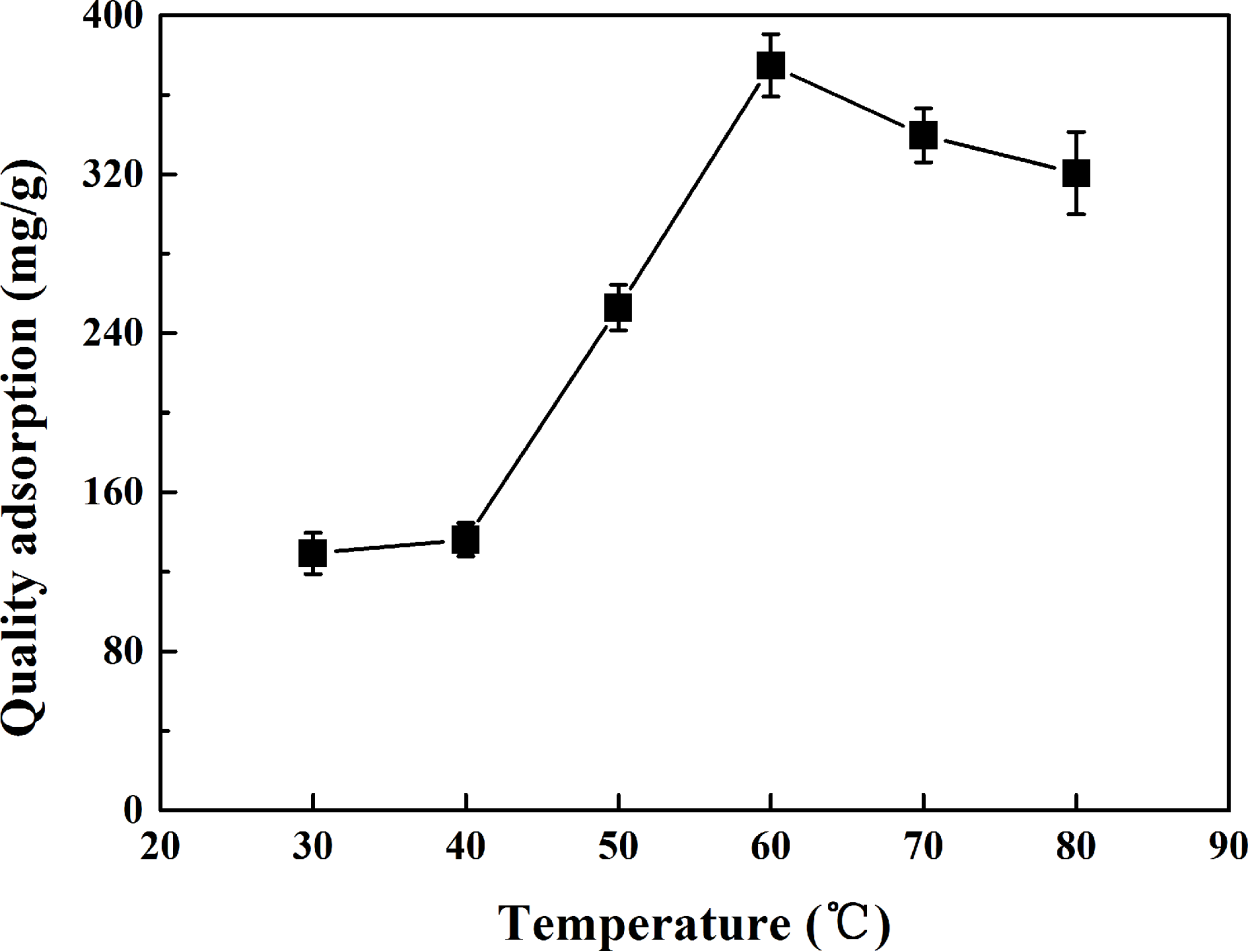
The effect of temperature on the adsorption of CMK-3.

Temperature was an important parameter in the adsorption process. Temperature not only affects the diffusion of PhGs molecules at the external boundary layer interface, but also inside the adsorbent pores. Figure 7 showed the effect of temperature on the adsorption capacity of CMK-3. The adsorption capacity of CMK-3 increase with temperature increased from 30 to 60°C. It has been reported the main reason was that the active site increases with increasing temperature duo to endothermic nature of the process, and the intra-particle diffusion of the adsorbents increased with the increase of the adsorption temperature (Peng et al., 2015). In addition, the mobility of the PhGs molecules increased and their diffusion resistance decreased with increasing temperature. The adsorption capacity decreases with increasing temperature when the temperature was 60–80°C. It indicated that the adsorption temperature had an optimum value and PhGs molecules may be instability at high temperatures. Therefore, the optimum adsorption temperature was chosen to be 60°C.

The optimum adsorption conditions of CMK-3 were as follows: the sample concentration was 0.41 mg/g, solution pH was 6, and adsorption temperature was 60°C. Under optimal condition, the adsorption capacity of PhGs in crude extract on the CMK-3 was 358.09 ± 4.13 mg/g, which was more than three times the adsorption capacity of PhGs on macroporous resins (HPD300, 94.93 mg/g) (Liu et al., 2013). Meanwhile, desorption rate of CMK-3 was 94.67% higher than that of macroporous resin.

### Adsorption Isotherms

**Figure 8:**
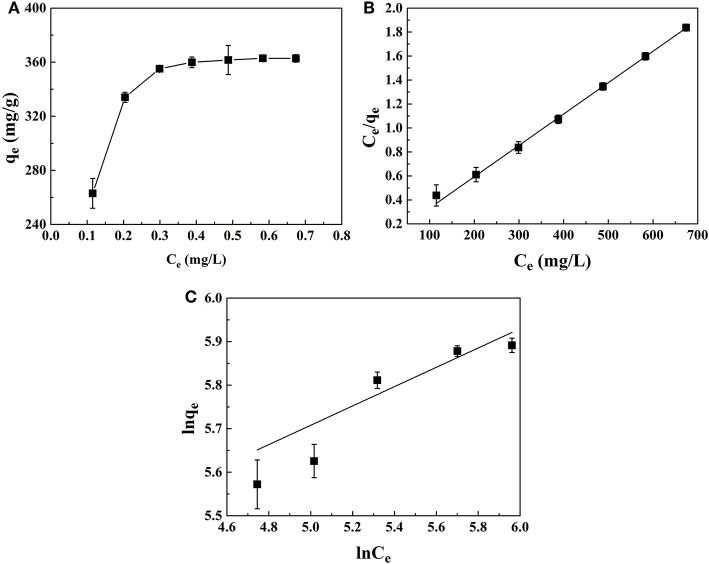
**(A)** Adsorption Isotherms **(B)** Langmuir and **(C)** Freundlich adsorption isotherm of PhGs on CMK-3.

The adsorption isotherms were shown in Figure 8A. With the increase of equilibrium concentration, the adsorption capacity for PhGs increased and reached saturation status. To further understand the adsorption performance of PhGs on CMK-3, the adsorption isotherms of CMK-3 were investigated using Langmuir and Freundlich models. The parameters of adsorption was obtained from different models provide some useful information on the adsorption mechanisms. Figures 8B,C depicted the CMK-3 adsorption isotherms that modeled by Langmuir and Freundlich model.

The Langmuir model was based on the assumptions that adsorption takes place at specific homogeneous sites within the adsorbent, no significant interaction occurs among adsorbed species, and the adsorbent was saturated after one layer of adsorbent molecules forms on the adsorbent surface. The linearized Langmuir isotherm equation can be written as follows:

(2) $$\frac{{\text{c}}_{\text{e}}}{{\text{q}}_{\text{e}}}=\frac{\text{1}}{{\text{K}}_{\text{L}}{\text{q}}_{\text{m}}}+\frac{{\text{c}}_{\text{e}}}{{\text{q}}_{\text{m}}}$$

The Freundlich model was commonly used to describe the adsorption characteristics of multilayer and heterogeneous surfaces (Wu et al., 2017). Its linearized form is given as follows:

(3) $${\text{lnq}}_{\text{e}}={\text{lnK}}_{\text{F}}+\frac{1}{\text{n}}{\text{lnc}}_{\text{e}}$$

where q_m_ (mg/g) is the theoretical maximum monolayer adsorption capacity, K_L_ (mL/mg) is the Langmuir constant related to the adsorption energy reflecting the affinity between the adsorbate and adsorbent (Wu et al., 2017; He et al., 2019), K_F_ [(mg/g)∙(mL/mg)^1/n^] and n are the Freundlich constants. K_F_ is an indicator of the relative adsorption capacity, n is related to the magnitude of the adsorption driving force and heterogeneity of the binding sites, and 1/n indicates the favorability of the adsorption.

The parameters of the isotherm models were summarized in Table 3. The Langmuir model was more better than the Freundlich model for describing the adsorption data of PhGs on CMK-3, which indicated that the adsorption of the PhGs on the CMK-3 was a simple monolayer adsorption process (Wang F. et al., 2017). In addition, the presence of oxygen-containing functional groups on the surface of CMK-3 enhanced the adsorption of PhGs. The maximum adsorption amount (q_m_) can reach 380.70 mg/g that was similar to the experimental value. In addition, the 1/n value of PhGs was calculated to be 0.22, which was <0.5. It indicated that the adsorption of the PhGs on the CMK-3 could take place easily (Fu et al., 2007; Gan et al., 2018). This indicated that the adsorption of PhGs on CMK-3 was not completely a physical adsorption process. PhGs contain multiple hydroxyl groups which might form hydrogen bonds with the oxygen-containing functional groups on the CMK-3 and offer weak chemical adsorption for the adsorption. Therefore, CMK-3 adsorption of PhGs was a complex adsorption process combining physical adsorption with chemisorption.

**Table 3:** Model parameters for adsorption of PhGs on CMK-3.

**Langmuir adsorption isotherm** ** (*****n*** **=** **5)**			**Freundlich adsorption isotherm** ** (*****n*** **=** **5)**		
**K_**L**_**	**q_**e**_(mg/g)**	***R*^**2**^**	**K_**F**_**	**1/n**	***R*^**2**^**
0.038 ± 0.025	380.70 ± 16.23	0.999 ± 0.005	99.32 ± 20.46	0.22 ± 0.074	0.737 ± 0.086

### Adsorption Kinetics

**Figure 9:**
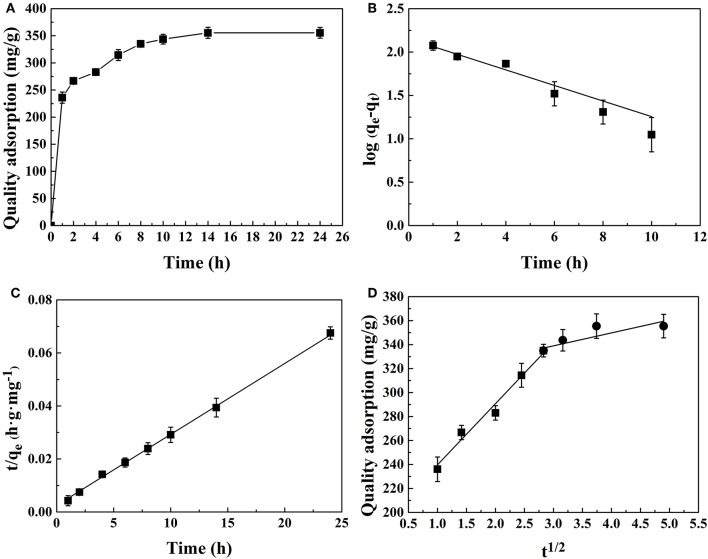
Adsorption kinetic models of PhGs onto CMK-3: **(A)** Adsorption kinetics of CMK-3 **(B)** pseudo-first-order, **(C)** pseudo-second-order, and **(D)** intra-particle diffusion model.

CMK-3 adsorption capacities were investigated as a function of time to determine the adsorption equilibrium time in Figure 9A. The investigation shows that the equilibrium adsorption rate gradually decreases and gradually leveled off as the adsorption capacity approaches equilibrium. It was found that the adsorption equilibrium was reached after 14 h. The rapid initial adsorption rate could be due to the high concentration gradient between the PhGs and CMK-3 in the solution and the surface of CMK-3 had large availability of active sites.

Adsorption kinetics was evaluated by the application of the pseudo-first-order, pseudo-second-order and intra-particle diffusion models. The plotted graphs of pseudo-first-order, pseudo-second-order models, and intra-particle diffusion model for adsorption PhGs onto CMK-3 were shown in Figures 9B–D, respectively.

The linear form of the kinetics models are expressed as follows:

Pseudo-first-order (Li et al., 2017a):

(4) $$\text{log}({\text{q}}_{\text{e}}-{\text{q}}_{\text{t}})={\text{logq}}_{\text{e}}-\frac{{\text{K}}_{1}}{2.303}\text{t}$$

Pseudo-second-order (Tang et al., 2018):

(5) $$\frac{\text{t}}{{\text{q}}_{\text{t}}}=\frac{1}{{\text{K}}_{2}{\text{q}}_{\text{e}}^{2}}+\frac{\text{t}}{{\text{q}}_{\text{t}}}$$

where K_1_ (1/min) and K_2_ (g/mg·min^−1^) are the rate constants of pseudo-first order rate equation and pseudo-second order rate equation, respectively. q_e_ (mg/g) is the theoretical adsorption capacity at equilibrium.

Intra-particle diffusion model:

(6) $${\text{q}}_{\text{t}}={\text{K}}_{\text{p}}{\text{t}}^{\frac{1}{2}}+\text{C}$$

where K_p_ is the intra-particle diffusion constant (mg/g min) and C is the reflection of boundary layer effect (mg/g).

The calculated values of q_e_, rate constants and correlation coefficient are shown in Table 4. The pseudo-second-order model gave an *R*^2^ value of 0.998, while *R*^2^ value of pseudo-first-order model was 0.84. It implies that the pseudo-second-order model exhibits better linear relationship than the pseudo-first-order model. Figure 9C shows that the linear plots of t/q_e_ vs. t show good agreement with the experimental data from the pseudo-second-order model. The calculated q_e_ of pseudo-second-order was quite similar to experimental data (Table 4). These results indicated that pseudo-second-order was more suitable kinetics model than pseudo-first-order.

**Table 4:** Parameters for the kinetic model of PhGs on CMK-3.

**Pseudo-first-order model** ** (*****n*** **=** **5)**			**Pseudo-second-order model** ** (*****n*** **=** **5)**		
**K_**1**_**	**q_**m**_(mg/g)**	***R*^**2**^**	**K_**2**_**	**q_**m**_(mg/g)**	***R*^**2**^**
0.090 ± 0.043	8.614 ± 3.62	0.84 ± 0.006	0.003 ± 0.001	373.13 ± 12.26	0.998 ± 0.005

However, the evaluation results of the pseudo-second-order model cannot determine the potential adsorption mechanism. It was generally accepted that adsorption kinetics was controlled by the diffusion mechanism, which consists external diffusion, boundary layer diffusion and intra-particle diffusion (Wong et al., 2019). The intra-particle diffusion model was used to determine if intra-particle diffusion were the rate-limiting steps. The intra-particle diffusion was said to be the rate limiting step when the q_t_ vs. t^1/2^ was linear. The intra-particle diffusion was only rate controlling step when the curve passes through the origin. It can be seen from Figure 9D, the intra-particle diffusion was governed by two different stages. The first stage of the curve represents surface adsorption. The second stage indicates intra-particle diffusion in the CMK-3 pores. As the intra-particle diffusion plot did not pass through the origin, the model indicated that the adsorption mechanism was more than one mechanism and intra-particle diffusion was not the only rate-limiting step. Thus, it can be concluded that the mechanism of PhGs adsorption on CMK-3 was complex that both the external surface adsorption and intra-particle diffusion occurred simultaneously.

### Repetitive Experiment

**Figure 10:**
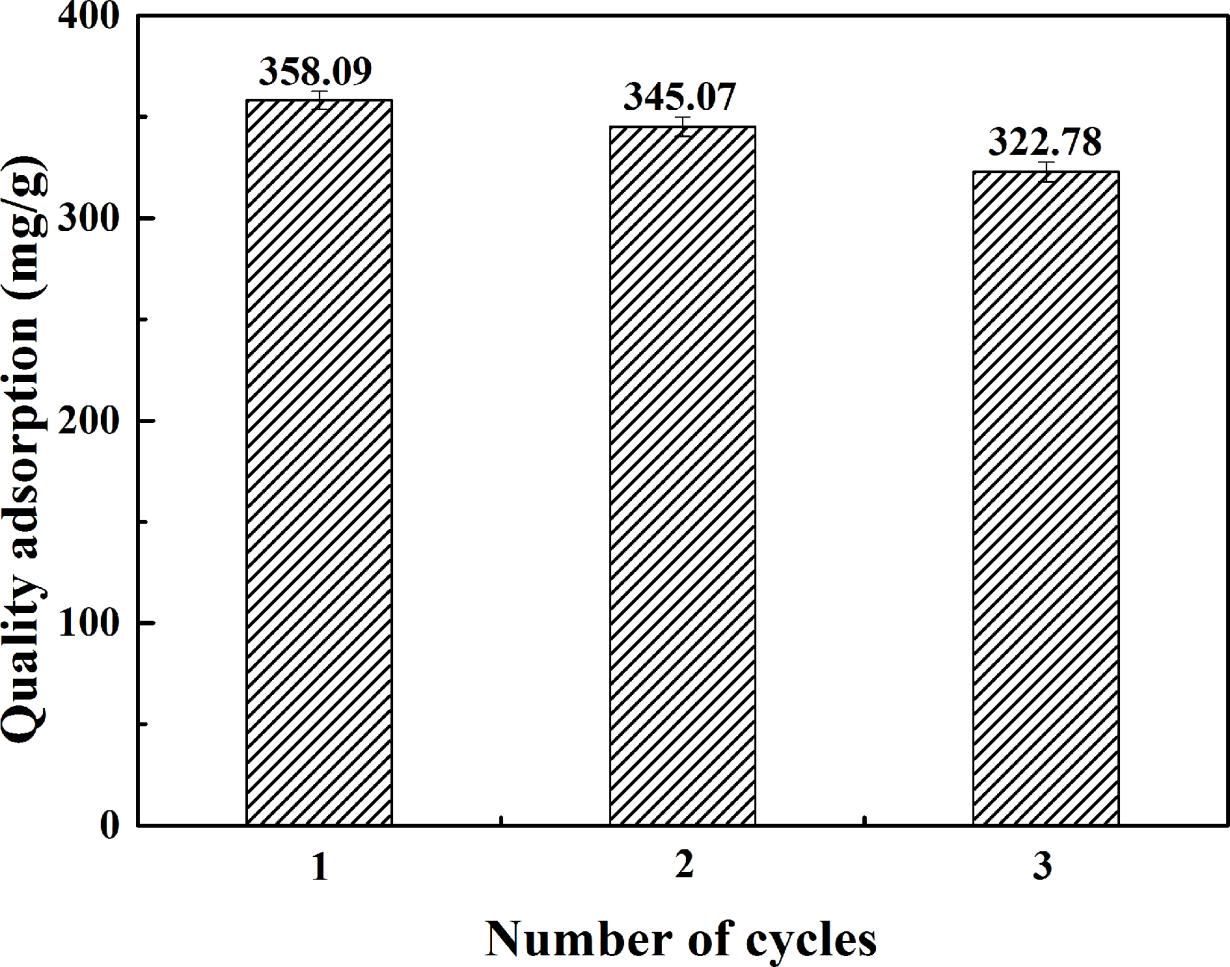
Cycle adsorption performance test of CMK-3.

Reusability was an important factor for considering the use and value of adsorbents in practical applications. Therefore, the cyclic adsorption performance of CMK-3 for the crude extract was tested. Figure 10 showed the results of cyclic adsorption of CMK-3 for crude extract of *Cistanche tubulosa*. It can be observed from the Figure 10 that the adsorption capacity of CMK-3 changed from 358.09 ± 4.13 mg/g to 320.78 ± 5.62 mg/g after three cycles of adsorption, which indicated the CMK-3 had good repeatability, and CMK-3 can be repeatedly used to adsorb PhGs.

## Conclusions

The adsorption properties of PhGs on the three kinds of mesoporous carbons were investigated and the mesoporous carbon before and after adsorption were characterized. The results showed that CMK-3 had a largest specific surface area and pore volume among the three adsorbents (CMK-3, DMC, and CMK-8), and it can adsorb PhGs molecules more effective than DMC and CMK-8 from the extracts of *Cistanche tubulosa*. Because the oxygen-containing functional groups on the surface of CMK-3 can provide a large number of active adsorption sites for the PhGs molecules. In addition, hydrogen bonds were formed between hydroxyl groups of PhGs and the oxygen-containing functional groups of CMK-3. The adsorption capacity of crude extract for PhGs was 358.09 ± 4.13 mg/g at the optimal conditions of 0.41 mg/L, pH = 6 and 60°C, and the corresponding desorption rate of CMK-3 was 95.02%. The adsorption data exhibited that adsorption of PhGs closely followed the Langmuir model and pseudo-second-order models, the intra-particle diffusion model suggested that the rate-limiting steps of adsorption were intra-particle diffusion model. The CMK-3 can be used as a potential adsorbent to highly efficient adsorb PhGs from *Cistanche tubulosa*.

## Data Availability Statement

All datasets generated for this study are included in the article/supplementary material.

## Author Contributions

HX carried out all the experiments, did data collection, data analysis, and wrote the manuscript. WP helped to analyze the FT-IR data and did data collection. XL helped to explain some of the experimental results and revise the manuscript. JZ contributed to the scientific interpretation of results and revised the manuscript.

### Conflict of Interest

The authors declare that the research was conducted in the absence of any commercial or financial relationships that could be construed as a potential conflict of interest.
